# A single G10T polymorphism in HIV-1 subtype C Gag-SP1 regulates sensitivity to maturation inhibitors

**DOI:** 10.1186/s12977-021-00553-5

**Published:** 2021-04-09

**Authors:** Dibya Ghimire, Yuvraj KC, Uddhav Timilsina, Kriti Goel, T. J. Nitz, Carl T. Wild, Ritu Gaur

**Affiliations:** 1 Faculty of Life Sciences and Biotechnology, South Asian University, New Delhi, 110021 India; 2 DFH Pharma, Gaithersburg, MD 20886 USA; 3 Present Address: Department of Microbiology and Immunology, University at Buffalo, Buffalo, NY 14203 USA

**Keywords:** HIV-1 subtype C, Maturation inhibitors, HIV-1 maturation, Resistant mutation, Polymorphism

## Abstract

**Background:**

Maturation inhibitors (MIs) potently block HIV-1 maturation by inhibiting the cleavage of the capsid protein and spacer peptide 1 (CA-SP1). Bevirimat (BVM), a highly efficacious first-in-class MI against HIV-1 subtype B isolates, elicited sub-optimal efficacy in clinical trials due to polymorphisms in the CA-SP1 region of the Gag protein (SP1:V7A). HIV-1 subtype C inherently contains this polymorphism thus conferring BVM resistance, however it displayed sensitivity to second generation BVM analogs.

**Results:**

In this study, we have assessed the efficacy of three novel second-generation MIs (BVM analogs: CV-8611, CV-8612, CV-8613) against HIV-1 subtype B and C isolates. The BVM analogs were potent inhibitors of both HIV-1 subtype B (NL4-3) and subtype C (K3016) viruses. Serial passaging of the subtype C, K3016 virus strain in the presence of BVM analogs led to identification of two mutant viruses—Gag SP1:A1V and CA:I201V. While the SP1:A1V mutant was resistant to the MIs, the CA:I120V mutant displayed partial resistance and a MI-dependent phenotype. Further analysis of the activity of the BVM analogs against two additional HIV-1 subtype C strains, IndieC1 and ZM247 revealed that they had reduced sensitivity as compared to K3016. Sequence analysis of the three viruses identified two polymorphisms at SP1 residues 9 and 10 (K3016: N9, G10; IndieC1/ZM247: S9, T10). The N9S and S9N mutants had no change in MI-sensitivity. On the other hand, replacing glycine at residue 10 with threonine in K3016 reduced its MI sensitivity whereas introducing glycine at SP1 10 in place of threonine in IndieC1 and ZM247 significantly enhanced their MI sensitivity. Thus, the specific glycine residue 10 of SP1 in the HIV-1 subtype C viruses determined sensitivity towards BVM analogs.

**Conclusions:**

We have identified an association of a specific glycine at position 10 of Gag-SP1 with an MI susceptible phenotype of HIV-1 subtype C viruses. Our findings have highlighted that HIV-1 subtype C viruses, which were inherently resistant to BVM, may also be similarly predisposed to exhibit a significant degree of resistance to second-generation BVM analogs. Our work has strongly suggested that genetic differences between HIV-1 subtypes may produce variable MI sensitivity that needs to be considered in the development of novel, potent, broadly-active MIs.

**Graphic abstract:**

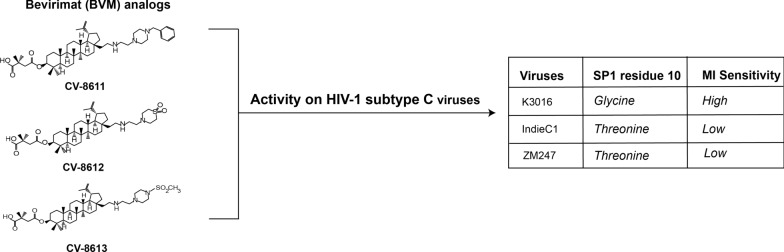

**Supplementary Information:**

The online version contains supplementary material available at 10.1186/s12977-021-00553-5.

## Background

There are 38 million people living with HIV/AIDS worldwide with 1.7 million new infections reported in 2019 [1]. The current treatment for AIDS involves Antiretroviral therapy (ART) comprising a combination of three or more distinct antiretroviral drugs. The U.S. Food and Drug Administration (FDA) has approved more than 25 antiretroviral drugs that are classified as entry or fusion inhibitors, nucleoside and nucleotide reverse transcriptase inhibitors (NRTI/NtRTI), non-nucleoside reverse transcriptase inhibitors (NNRTI), integrase inhibitors and protease inhibitors [2]. ART results in the improvement in the clinical care of HIV-infected patients, although complete elimination of the virus cannot be achieved. Moreover, long term usage of antiretroviral drugs can decrease the drug tolerability and lead to emergence of drug resistant viruses necessitating a continued requirement for the development of new anti-HIV drugs [3–5]. In this regard, maturation inhibitors (MIs) represent a promising novel class of anti-HIV compounds. HIV maturation involves a highly ordered sequential cleavage of the full-length Gag polyprotein by the viral protease to produce mature virions. This step is essential to produce the infectious particle [6]. Virus maturation can be inhibited by two distinct classes of Gag inhibitors, protease and maturation inhibitors. The protease (PR) inhibitors (PIs) bind to HIV-1 protease enzyme directly and inhibit its enzymatic activity. They compete with HIV Gag for binding to the active site of the protease enzyme [7]. There are currently 11 FDA-approved protease inhibitors that are being used in the treatment of AIDS patients worldwide [8]. Whereas, the maturation inhibitors (MIs) bind to the HIV Gag protein instead and block the access of the protease enzyme to cleave Gag. Specifically, the cleavage of CA and SP1 is inhibited which is essential to produce mature capsid protein.

HIV-1 maturation occurs concomitantly or soon after virus release from cells in a highly orchestrated way involving sequential cleavage of the HIV Gag precursor to release matrix (MA), capsid (CA), nucleocapsid (NC) and p6 proteins [6, 9, 10]. The rate of cleavage varies at each step, with the cleavage of CA-SP1, the last and the slowest [10–12]. In the immature virus particle, the Gag precursor proteins are organized radially just below the envelope of the particle. Structurally, the immature HIV-1 capsid-C terminal domain-Spacer peptide1 (CA-CTD-SP1) Gag fragments form a hexameric structure that looks like a goblet, with the main CA-CTD domain forming the cup and the stem consists of compactly folded CA-CTD-SP1 junction helices. The compact stem region sequesters the CA-SP1 cleavage site making it inaccessible to the HIV protease (PR) until the 6-helix bundle unfolds. Hence, the last step is the rate-limiting step in the HIV-1 maturation process [13, 14] and is required for the formation of the conical core of the capsid and governs viral infectivity [6]. MIs stabilize the immature HIV-1 Gag lattice by inhibiting the unfolding of the 6-helix bundle [15], thereby preventing viral protease-mediated cleavage of CA-SP1 and resulting in an immature and noninfectious virus [16, 17].

The first-in-class maturation inhibitor, Bevirimat (BVM) (a betulinic acid derivative), reduced cleavage of CA from SP1 leading to accumulation of the CA-SP1 intermediate [18–20]. BVM demonstrated potency against both wild type and drug-resistant HIV-1 isolates in vitro but failed in Phase 2B clinical trials owing to natural polymorphisms present in the SP1 region of Gag, specifically those present in the “QVT” motif at SP1 residues 6–8 [21, 22]. HIV-1 subtype C viruses, contribute to nearly 50% of the total HIV-1 infections worldwide and are predominant in South Asian countries including India, China and Eastern and Southern Africa [23]. Subtype C viruses contain naturally occurring polymorphisms in the SP1 QVT motif and are resistant to BVM [17, 23, 24]. BVM analogs with modifications at C-3, C-28 or C-30 positions displayed enhanced potency and broad activity against multiple HIV strains including HIV-1 subtype C unlike the parental BVM [16, 17, 25–29]. The GSK3532795 (formerly BMS955176) compound carrying modifications at C-3 and C-28 positions of BVM, entered Phase 2B trials but was discontinued due to higher rates of gastrointestinal intolerability and the emergence of resistant viruses [30, 31]. On the other hand, another second-generation MI, GSK2838232, had demonstrated promising results in Phase IIA clinical trial. Co-administration of GSK2838232 with cobicistat demonstrated antiviral efficacy as a short-term monotherapy in participants infected with HIV-1. The treatment was safe and well tolerated without any adverse events [32, 33]. Another second-generation MI, GSK3640254, was evaluated to be safe and efficacious in two phase I clinical trials [34].

In this study, we have tested the activity of three novel second-generation C-28 alkyl amine derivatives of BVM (CV-8611, CV-8612 and CV-8613) against multiple HIV-1 subtype B and C strains. All the compounds inhibited CA-SP1 processing and reduced viral infectivity against HIV-1 subtype B but displayed differential activity against HIV-1 subtype C molecular clones (K3016, IndieC1 and ZM247). The K3016 clone was more sensitive to BVM analogs than both IndieC1 and ZM247 due to the presence of polymorphism at SP1 residue 10.

## Results

### BVM analogs inhibit HIV-1 subtype B and C viruses

**Fig. 1:**
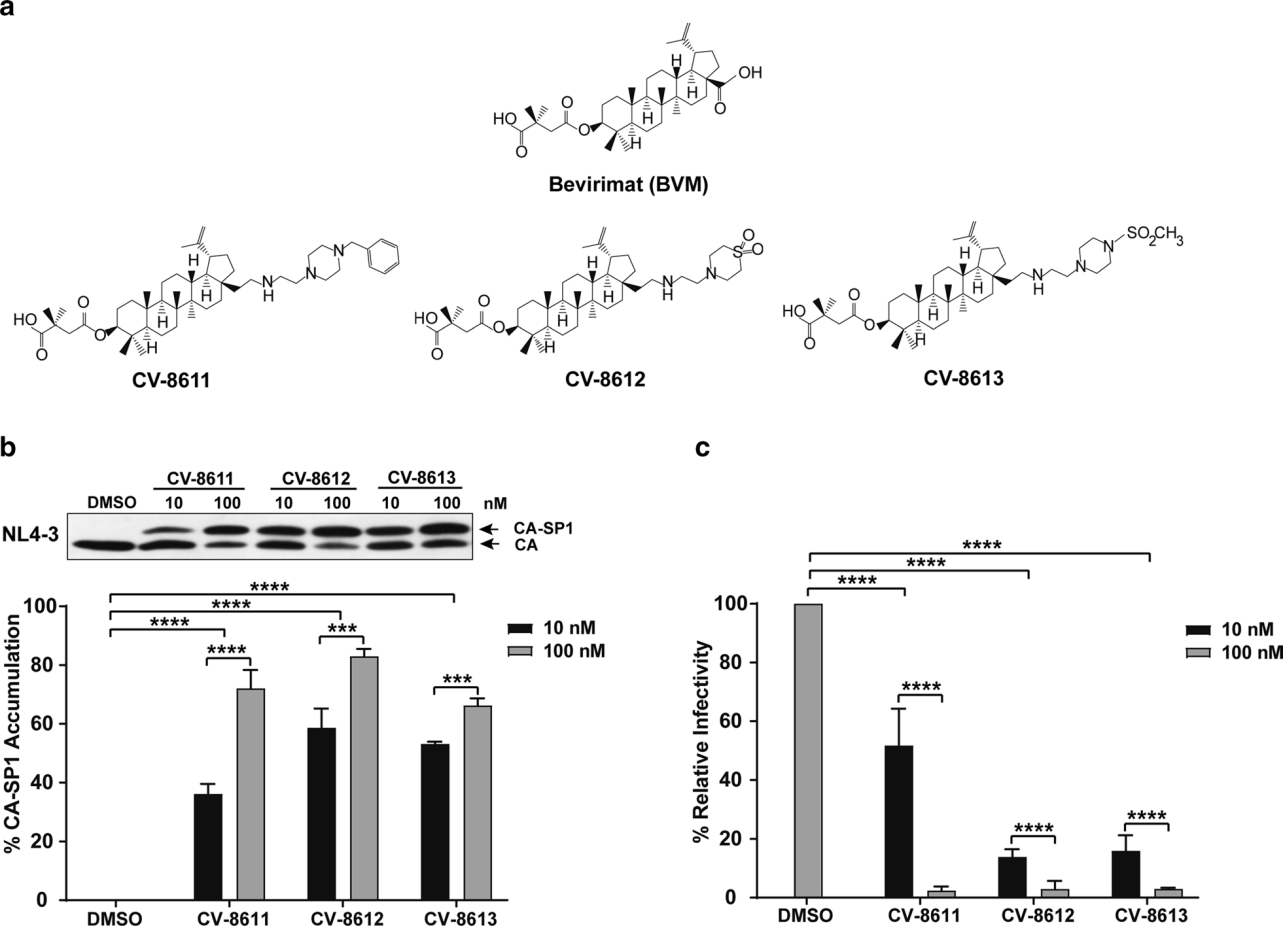
BVM analogs are active against HIV-1 subtype B, NL4-3. **a** Structure of parental compound Bevirimat (BVM) and its C-28 modified analogs CV-8611, CV-8612, and CV-8613. HEK-293T cells were transfected with the HIV-1 subtype B clone NL4-3. Cells were treated with 10 nM and 100 nM of the BVM analogs or with DMSO only. **b** The virion-associated CA and CA-SP1 were detected by immunoblotting. The gel image shown here is representative of three independent experiments. Quantification of % CA-SP1 relative to total CA + CA-SP1 is presented in the graphs. **c** The viruses produced from MI-treated HEK-293 T cells were quantified. TZM-bl cells were infected with p24 normalized HIV-1 viruses for 48 h. The cells were lysed and assayed for luciferase activity. Quantitative data for levels of infectivity relative to the DMSO control-treated sample is shown. Error bars indicate standard deviations from three independent experiments. The asterisks indicate significant differences (*p < 0.05, **p < 0.01, ***p < 0.001, ****p < 0.0001) and ns indicates not significant

Several C-28 analogs of BVM have been reported to be active against the HIV-1 subtype B NL4-3 virus, the NL4-3 SP1 V7A mutant, multiple HIV-1 primary isolates, and HIV-1 subtype C viruses [16, 17]. Three novel BVM analogs (CV-8611, CV-8612 and CV-8613) were synthesized by introducing alkyl amine modifications at the C-28 position of BVM (Fig. 1a). We tested the activity of the BVM analogs against the HIV-1 subtype B, NL4-3 and the HIV-1 subtype C, K3016 viral strain. HEK-293T cells were transfected with a plasmid DNA encoding the HIV-1 subtype B or C virus in the absence or presence of two different concentrations of the BVM analogs (10 nM and 100 nM) as described in the Methods. The virus lysates were analyzed for accumulation of the CA-SP1 intermediate (p25) (Fig. 1b). There was a 65–83% increase in the CA-SP1 intermediate in the presence of 100 nM of the compounds suggesting that the BVM analogs inhibited HIV-1 subtype B Gag processing (Fig. 1b). The infectivity of the viruses produced in the presence of the compounds was measured by a TZM-bl cell-based single-cycle infectivity assay, as explained in “Methods”. This assay is based on the expression of a luciferase reporter gene, under the control of the LTR promoter and is considered a sensitive and quantitative measure of virus infection [35]. In the presence of 100 nM compounds, there was a 95% reduction in the infectivity of the HIV-1 subtype B virus confirming that the second generation BVM analogs potently inhibited the HIV-1 subtype B (Fig. 1c).

**Fig. 2:**
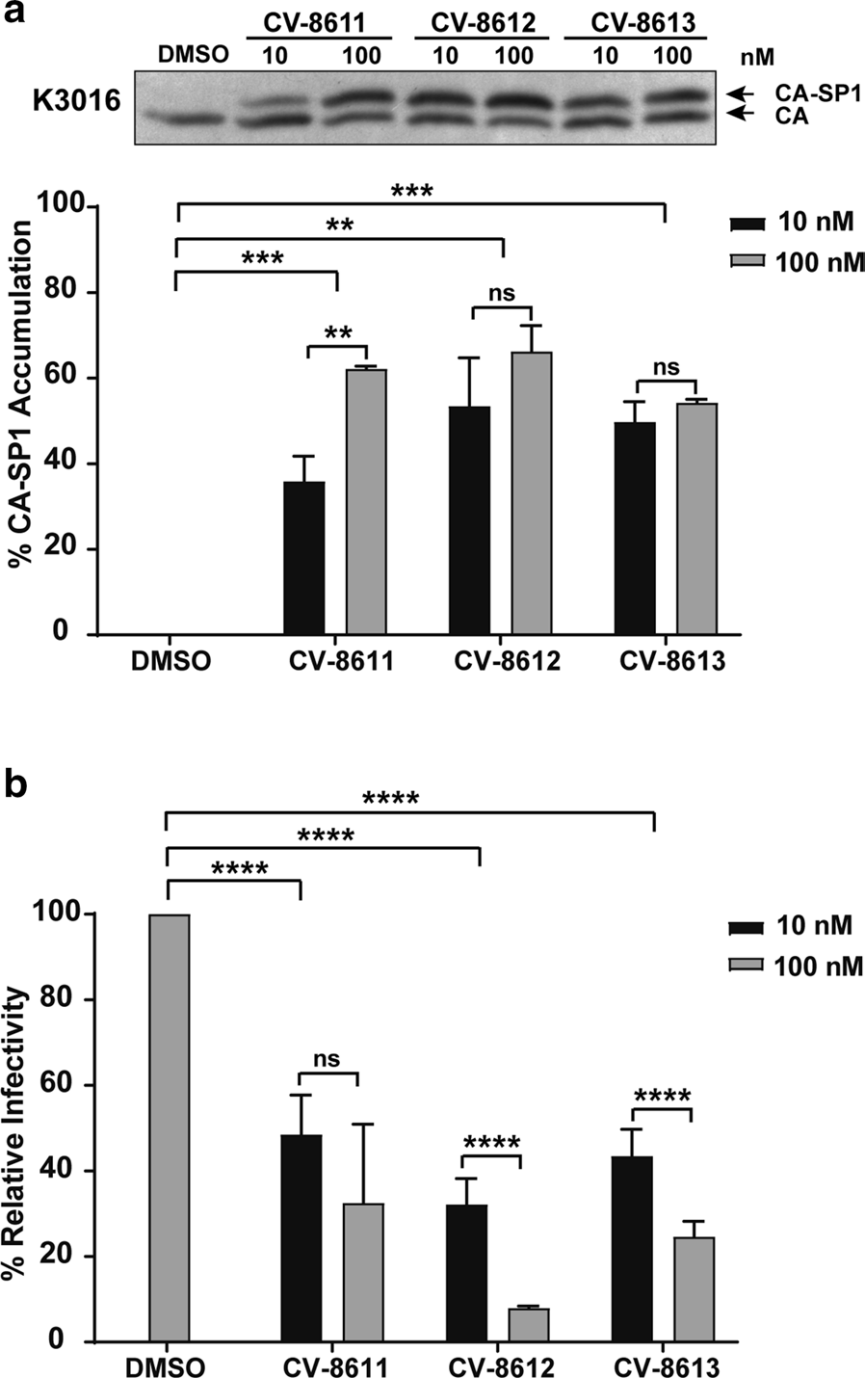
BVM analogs are active against HIV-1 subtype C K3016. HEK-293T cells were transfected with the HIV-1 subtype C molecular clone K3016. Cells were treated with 10 nM and 100 nM of the BVM analogs or with DMSO only. **a** The virion-associated CA and CA-SP1 were detected by immunoblotting. The gel image shown here is representative of three independent experiments. Quantification of % CA-SP1 relative to total CA + CA-SP1 is presented in the graphs. **b** The viruses produced from MI-treated HEK-293T cells were quantified, and TZM-bl cells were infected with p24 normalized HIV-1 viruses for 48 h. The cells were lysed and assayed for luciferase activity. Quantitative data for levels of infectivity relative to the DMSO control-treated sample is shown. Error bars indicate standard deviations from three independent experiments. The asterisks indicate significant differences (*p < 0.05, **p < 0.01, ***p < 0.001, ****p < 0.0001) and ns indicates not significant

To determine whether the new BVM analogs displayed broad specificity against other HIV-1 strains, we tested their activity in inhibiting the CA-SP1 cleavage and viral infectivity of the HIV-1 subtype C molecular clone, K3016. We observed an accumulation of the CA-SP1 intermediate (55–65%) in the presence of 100 nM of the BVM analogs (Fig. 2a). Furthermore, the viruses produced in the presence of these analogs displayed a 75–90% reduction in the infectivity at 100 nM concentration (Fig. 2b). These results demonstrated that the novel BVM analogs were broadly active against both HIV-1 subtype B and C strains.

We next determined the antiviral activity of the BVM analogs against K3016 virus by measuring their IC_50_ values as described in the Methods. The antiviral activity was calculated by measuring the reduction in HIV-1 p24 concentration in the culture supernatants. The IC_50_ values of all the compounds was calculated to be in the low nanomolar range with lowest IC_50_ observed with CV-8613 (Table 1).

**Table 1:** Antiviral activities of the BVM analogs against the HIV-1 subtype C K3016 virus

Compound	IC_50_ (nM)
CV-8611	3.89 ± 0.18
CV-8612	3.60 ± 0.22
CV-8613	2.27 ± 0.56

The IC_50_s represent the means ± standard deviations from two independent experiments

### BVM analogs delayed replication of the HIV-1 subtype C virus

**Fig. 3:**
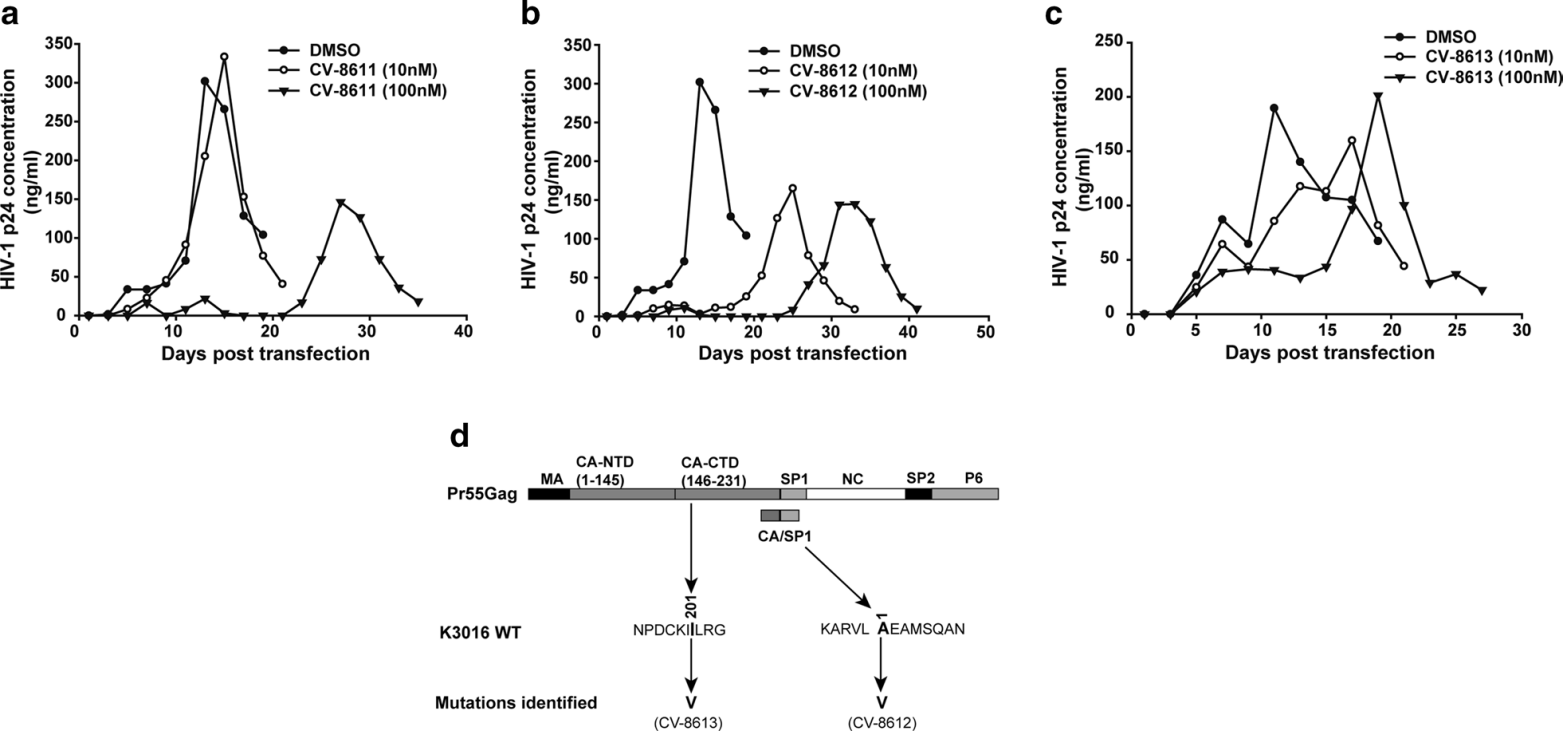
BVM analogs delays HIV-1 subtype C K3016 replication. The HUT-R5 T-cells were transfected with the K3016 molecular clone. Transfected cells were proliferated in the presence of 10 nM and 100 nM of **a** BVM analog CV-8611 or with DMSO only, **b** BVM analog CV-8612 or with DMSO only and **c** BVM analog CV-8613 or with DMSO only. Virus replication was observed by quantifying HIV-1 p24 concentration in the culture supernatant, and the replication profile was represented as a graph. **d** The HUT-R5 T-cells were transfected with K3016 molecular clone and propagated in the presence of 100 nM of the BVM analogs (CV-8611, CV-8612, and CV-8613). The virus produced was re-passaged multiple times in HUT-R5 T-cells. The Gag and Protease coding region of the HIV-1 virus was sequenced to analyze the resistance profile. The figure depicts the sites of the mutation

Next, replication efficiency of the HIV-1 subtype C K3016 virus in the presence of BVM analogs was tested as described in the Methods. Briefly, HUT-R5 T-cells were transfected with the K3016 molecular clone in the presence of two different concentrations of BVM analogs (10 nM and 100 nM). Virus replication kinetics was monitored by quantifying the HIV-1 p24 antigen in the culture supernatant. The K3016 virus peaked at day 13 in the absence of the compounds, but with a significant delay in the presence of all the compounds, CV-8611 (day 27), CV-8612 (day 33) and CV-8613 (day 21) at 100 nM (Fig. 3a–c). This delay suggested that the replication capacity of the K3016 WT virus was inhibited in the presence of all BVM analogs. We sequenced the entire Gag and protease encoding region of the virus at the peak replication day and identified two mutations acquired by the virus- SP1: A1V and CA: I201V (Fig. 3d).

### SP1: A1V mutant was resistant to the BVM analog

**Fig. 4:**
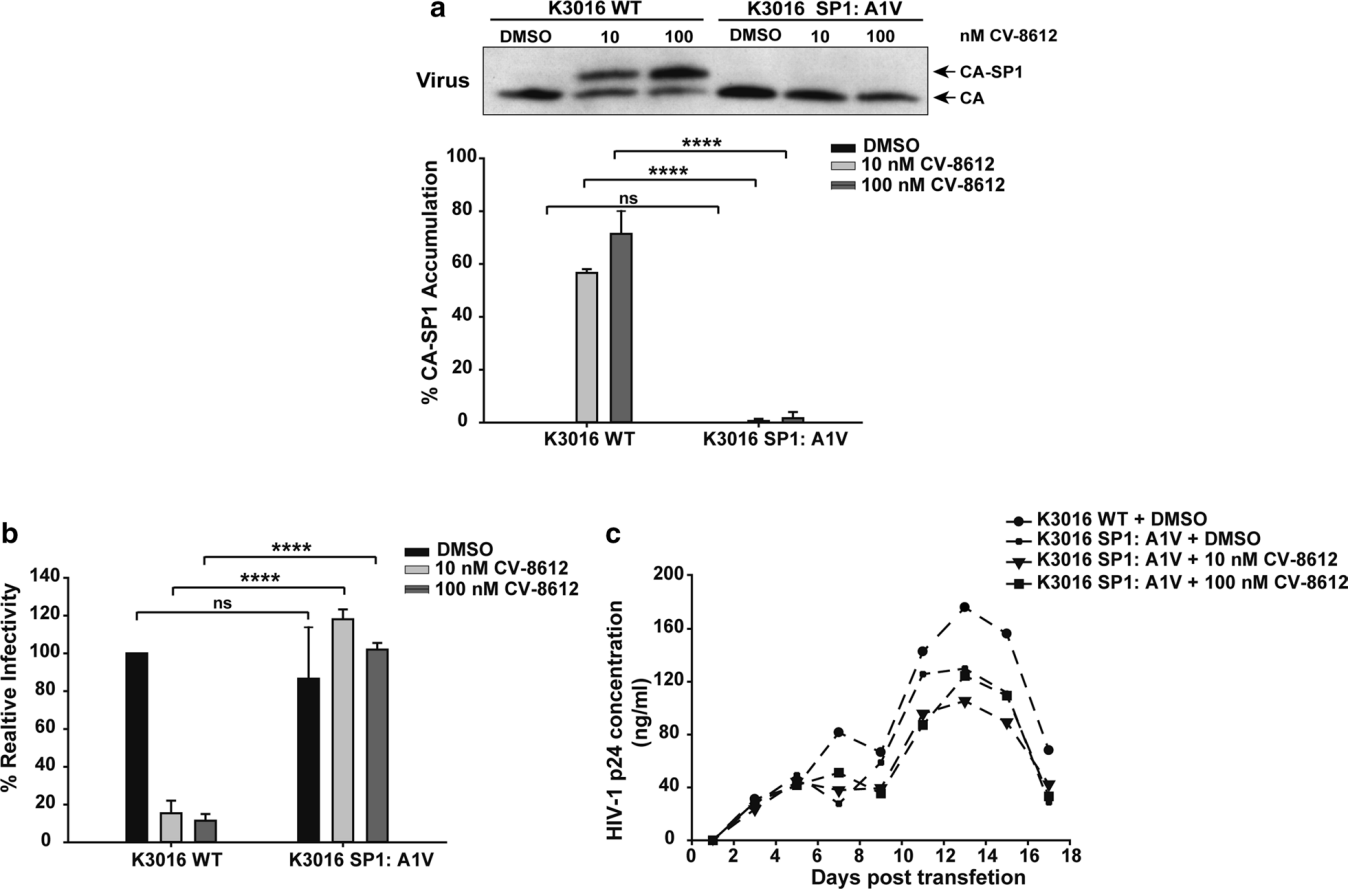
K3016 SP1: A1V mutant is resistant to BVM analog. HEK-293 T cells were transfected with the HIV-1 subtype C K3016 WT and K3016 SP1: A1V mutant. Cells were treated with 10 nM and 100 nM of BVM analog CV-8612 or with DMSO only. **a** The virion-associated CA and CA-SP1 were detected by immunoblotting. % CA-SP1 accumulation is presented in the graphs. The gel image shown here is representative of three independent experiments. **b** The viruses produced from the BVM analogs-treated HEK-293T cells were quantified, and TZM-bl cells were infected with p24 normalized viruses for 48 h. The cells were lysed and assayed for luciferase activity. Quantitative data for levels of infectivity relative to the DMSO control-treated sample is shown. Error bars indicate standard deviations from three independent experiments. The asterisks indicate significant differences (*p < 0.05, **p < 0.01, ***p < 0.001, ****p < 0.0001) and ns indicates not significant. **c** The HUT-R5 T-cells were transfected with the HIV-1 subtype C molecular clone K3016 WT and K3016 SP1: A1V mutant. Transfected cells were propagated in the presence of 10 and 100 nM of the BVM analog CV-8612 or with DMSO only. Virus replication was monitored by quantifying HIV-1 p24 concentration in the culture supernatant, and the replication profile was represented as a graph

We introduced the SP1:A1V mutation into the K3016 WT backbone to generate the mutant clone and characterized its resistance profile by analyzing the effect of this mutation on CA-SP1 processing, infectivity, and virus replication. Briefly, HEK-293T cells were transfected with the plasmid DNA encoding the HIV-1 subtype C K3016 WT and the mutant in the absence or presence of two concentrations of CV-8612 (10 nM and 100 nM). Unlike the WT, the mutant A1V did not display an accumulation of the CA-SP1 intermediate in the presence of the BVM analog (Fig. 4a). The mutant viruses produced in the presence of the compound retained infectivity unlike the WT in a single-round infectivity assay (Fig. 4b). In addition, the A1V mutant viruses displayed similar replication kinetics in the presence as well as the absence of the compound (Fig. 4c) confirming that the A1V mutation conferred resistance to the compound.

### CA: I201V mutation conferred partial resistance and compound dependent phenotype on the virus

**Fig. 5:**
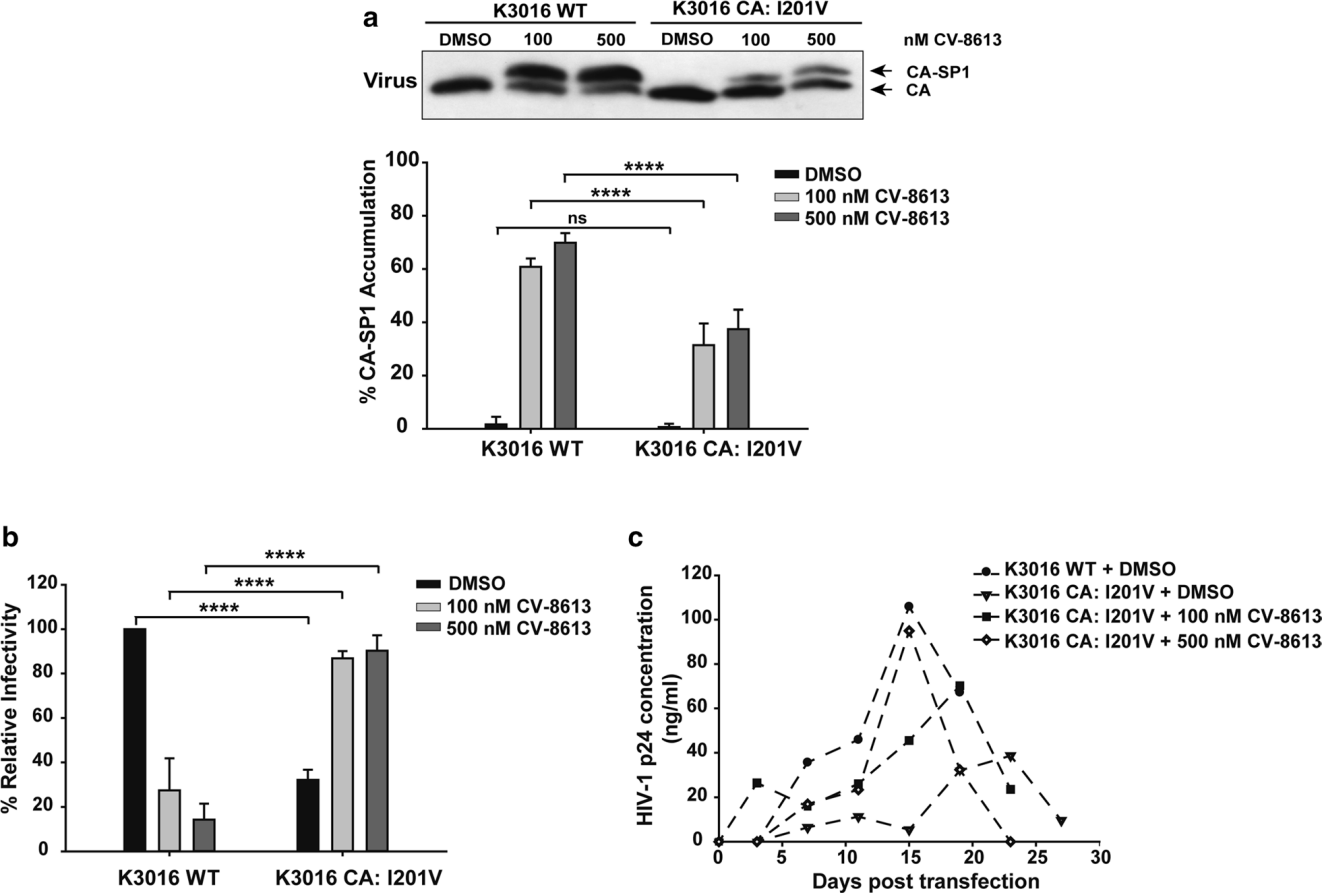
K3016 CA: I201V mutant is partially resistant and compound-dependent. HEK-293T cells were transfected with the HIV-1 subtype C K3016 WT and K3016 CA: I201V mutant and treated with 100 nM and 500 nM of the BVM-analog CV-8613 or with DMSO only. **a** The CA-SP1 accumulation assay was performed. Gel images shown here are representative of three independent experiments. Quantification of % CA-SP1 relative to total CA + CA-SP1 is presented in the graphs. **b** The viruses produced by BVM-analog-treated HEK-293T cells were quantified, and TZM-bl cells were infected with these MI-treated HIV-1 viruses for 48 h. The cells were lysed and assayed for luciferase activity. Quantitative data for levels of infectivity relative to the DMSO control-treated sample is shown. Error bars indicate standard deviations from three independent experiments. The asterisks indicate significant differences (*p < 0.05, **p < 0.01, ***p < 0.001, ****p < 0.0001) and ns indicates not significant. **c** The HUT-R5 T-cells were transfected with the HIV-1 Subtype C molecular clone K3016 WT and K3016 CA: I201V mutant. Transfected cells were propagated in the presence of 100 nM and 500 nM of the BVM-analog CV-8613 or with DMSO only. Virus replication was monitored by quantifying HIV-1 p24 concentration in the culture supernatant, and the replication profile was represented as a graph

To analyze the effect of the CA: I201V mutation, HEK-293T cells were transfected with the K3016 WT and the mutant virus in the absence or the presence of two concentrations of BVM analog CV-8613 (100 nM and 500 nM). The mutant virus displayed reduced accumulation of the CA-SP1 intermediate (30%) as compared to the WT (70%) in the presence of high concentration of the analog (500 nM) indicating that the mutation conferred partial resistance to the virus (Fig. 5a). The infectivity of the I201V mutant was significantly reduced by 70% in comparison to the WT in the absence of the analog (Fig. 5b). However, the infectivity of the mutant virus was almost completely rescued in the presence of the compound (Fig. 5b) suggesting that the I201V mutant gained infectivity in the presence of the compound.

A similar phenomenon was observed on studying the replication of the WT and the mutant virus. The WT virus peaked at day 13 whereas the mutant virus was less fit and exhibited a delay of 10 days and peaked at day 23 in the absence of the BVM analog CV-8613. The replication of the mutant improved in the presence of increasing concentration of the analog (Fig. 5c) and displayed kinetics similar to the WT at 500 nM concentration of the BVM analog. These results confirmed that the CA: I201V mutation conferred partial resistance and compound dependent phenotype on the virus.

### BVM analogs were less potent against other HIV-1 subtype C strains

**Fig. 6:**
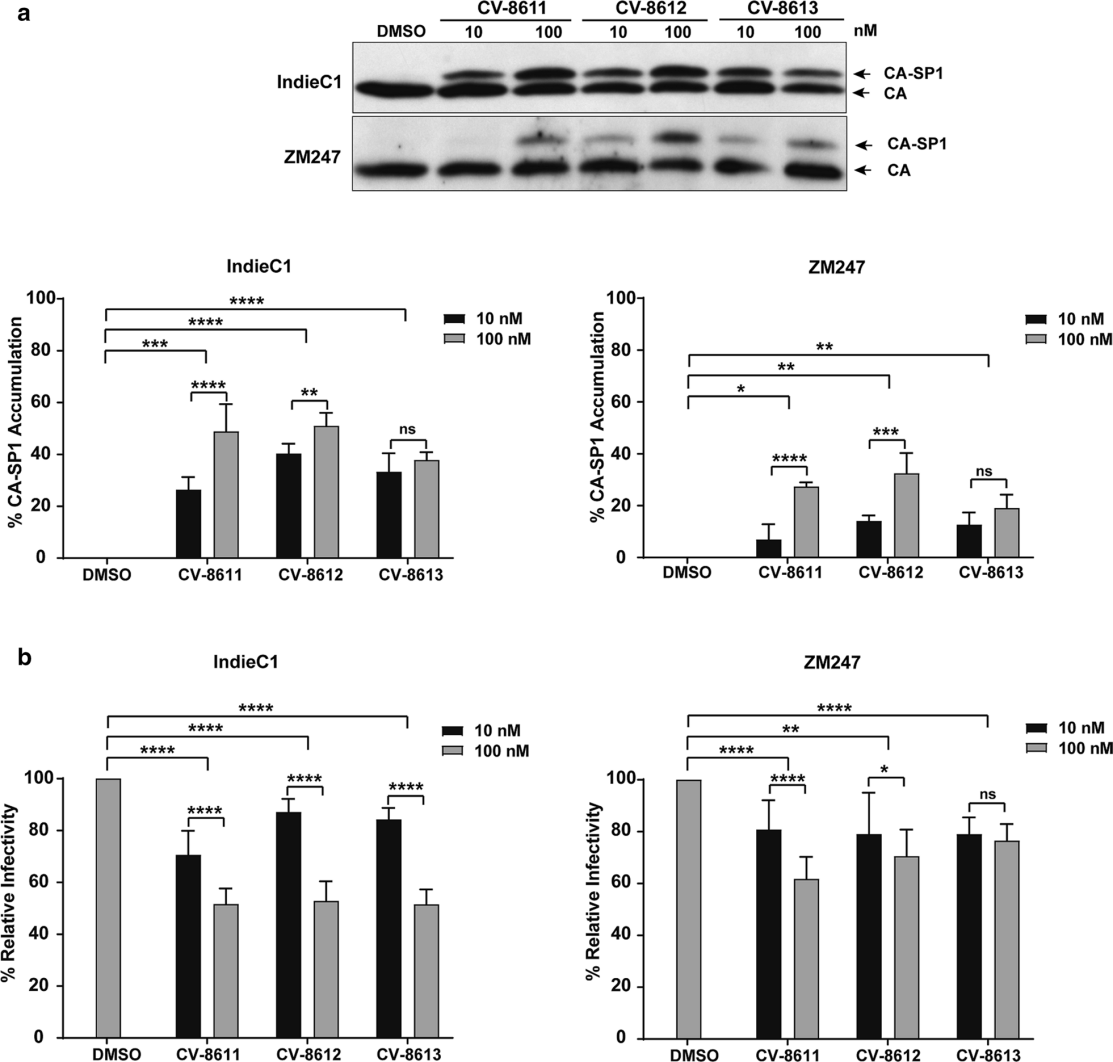
BVM analogs are less sensitive to HIV-1 subtype C viruses, IndieC1 and ZM247. HEK-293T cells were transfected with the HIV-1 subtype C molecular clone IndieC1 and ZM247. Cells were treated with 10 nM and 100 nM of the BVM analogs or with DMSO only. **a** The virion-associated CA and CA-SP1 were detected by immunoblotting. The gel image shown here is representative of three independent experiments. Quantification of % CA-SP1 relative to total CA + CA-SP1 is presented in the graphs. **b** The viruses produced from the BVM analog-treated cells were quantified, and TZM-bl cells were infected with these HIV-1 viruses for 48 h. The cells were lysed and assayed for luciferase activity. Quantitative data for levels of infectivity relative to the DMSO control-treated sample is shown. Error bars indicate standard deviations from three independent experiments. The asterisks indicate significant differences (*p < 0.05, **p < 0.01, ***p < 0.001, ****p < 0.0001) and ns indicates not significant

We tested the efficacy of the three BVM analogs against two additional HIV-1 subtype C molecular clones, IndieC1 and ZM247 by measuring their effect on p25 accumulation and infectivity (Fig. 6). Both IndieC1 and ZM247 were less sensitive to the BVM analogs as evident from reduced CA-SP1 accumulation as compared to K3016 (compare Figs. 6a with 2a). ZM247 was least sensitive to the BVM analogs and displayed only 25% CA-SP1 accumulation at 100 nM of the compounds (Fig. 6a). These analogs were also less effective in reducing the infectivity of both IndieC1 and ZM247 and the viruses retained significant infectivity even in the presence of the BVM analogs as compared to K3016 (compare Figs. 6b with 2b). These results suggested that the HIV-1 subtype C strains IndieC1 and ZM247 were less sensitive to second generation BVM analogs.

### A single amino acid in the SP1 region of Gag regulated sensitivity of HIV-1 subtype C to BVM analogs

**Fig. 7:**
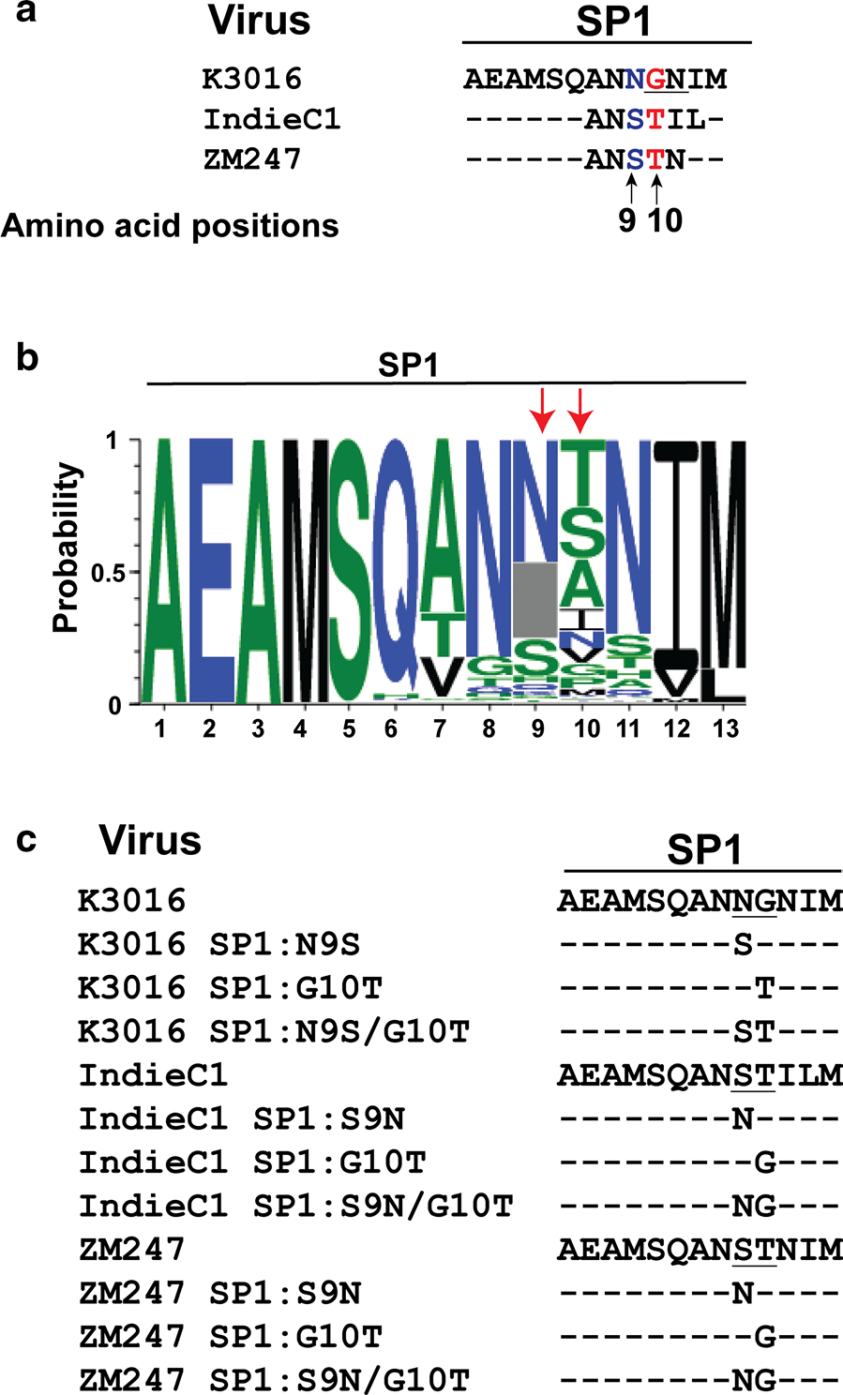
Sequence analysis and construction of mutants in K3016, IndieC1 and ZM247 viral backbone. **a** Amino acid sequence alignment of the SP1 region of the HIV-1 subtype C clones, K3016, IndieC1 and ZM247. **b** Sequence logos for HIV-1 subtype C (1994 sequences) SP1 residues derived from the curated Los Alamos HIV-1 sequence database. Positions of amino acid residues are numbered (X-axis). Conservation of a residue is shown as a function of probability of its occurrence at a particular position (Y-axis). Blue, green and black indicates hydrophilic, neutral and hydrophobic amino acids respectively. Red arrows indicate positions of amino acids selected for our study. **c** Construction of HIV-1 subtype C SP1 mutants (underlined). Dashes represent residues that are identical to the parental sequence

Next, we explored the possible mechanism behind the differential sensitivity of the HIV-1 subtype C viruses to the BVM analogs. It has been well documented that several amino acids in the SP1 region play a critical role in regulating the activity of MIs [22, 24, 28, 36–39]. On aligning the SP1 region of the three HIV-1 subtype C strains, we observed that the amino acids at position 9 and 10 were identical in IndieC1 and ZM247 but different in K3016 (Fig. 7a). Specifically, at the SP1 9th position, there is an asparagine (N) in K3016 while IndieC1 and ZM247 have a serine (S). Similarly, at the 10th position, a glycine (G) is present in K3016 as opposed to a threonine (T) in IndieC1 and ZM247 (Fig. 7a). We have previously reported that a mutation in the SP1 residue 10 in the K3016 virus conferred partial resistance to the BVM analog 7 m [38]. Analysis of 1194 HIV-1 subtype C viruses from the curated Los Alamos database revealed that 65% of the sequences contained asparagine and 20% serine at SP1:9 position. The SP1:10 residue on the other hand contained; 25.61% threonine and only 4.89% glycine (Fig. 7b). We hypothesized that these two amino acids may play a role in governing differential sensitivity of the viruses to the BVM analogs. We swapped the residues at SP1 position 9 and 10 between the HIV-1 subtype C strains by site-directed mutagenesis and constructed two single mutants K3016 SP1: N9S and K3016 SP1: G10T and a double mutant K3016 SP1: N9S/G10T. Similarly, two single mutants and a double mutant were constructed in the IndieC1 and ZM247 backbone by replacing serine with an asparagine in the 9^th^ position and glycine with a threonine in the 10th position (Fig. 7c).

**Fig. 8:**
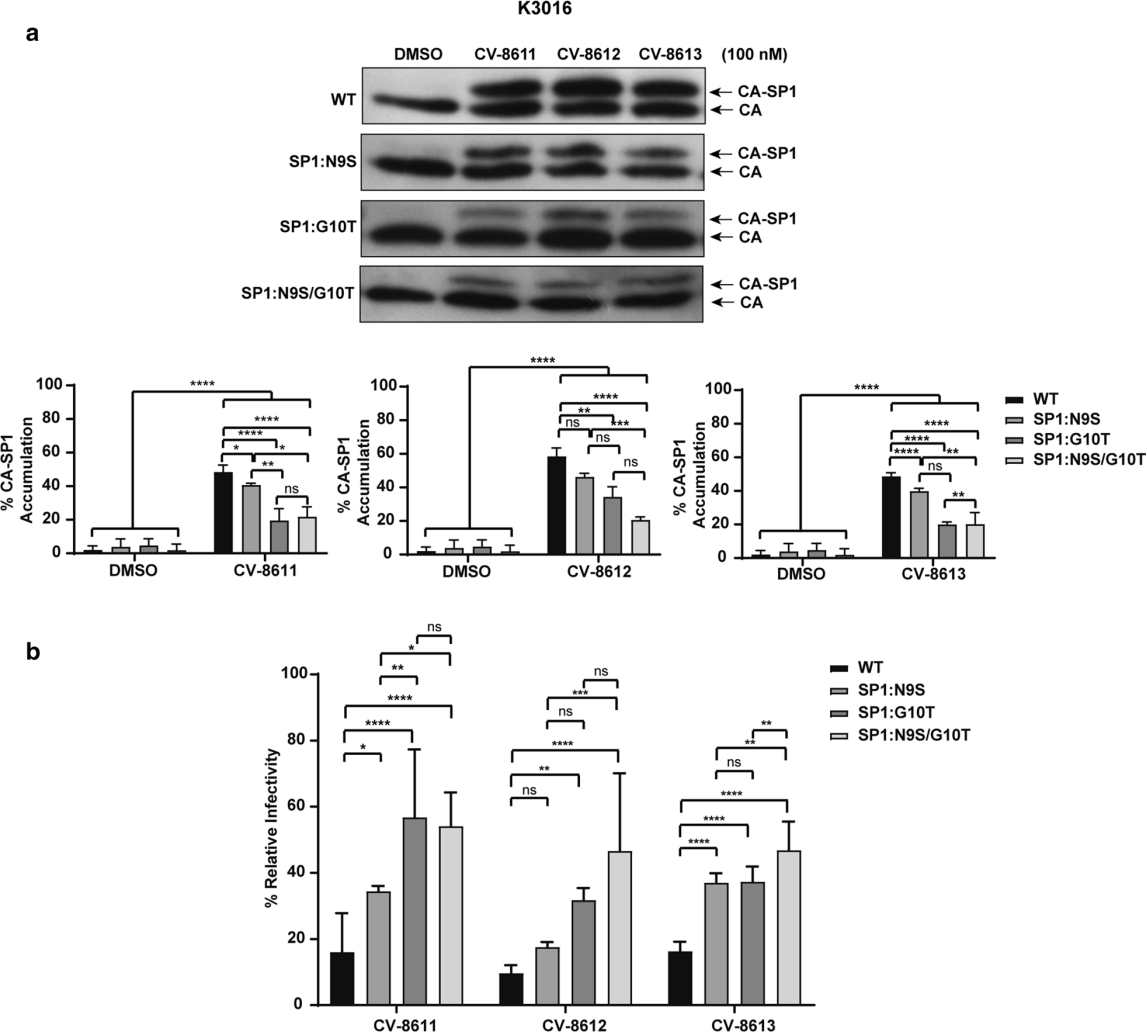
Mutation in HIV-1 subtype C K3016 Gag-SP1 residue 10 decreases sensitivity towards BVM analogs. **a** HEK-293 T cells were transfected with the HIV-1 subtype C clones, K3016 WT and its mutants SP1: N9S, SP1:G10T and SP1:N9S/G10T. Cells were treated with 100 nM of BVM analogs CV-8611, CV-8612, CV-8613 or with DMSO only. The virion-associated CA and CA-SP1 were detected by immunoblotting. Quantification of % CA-SP1 relative to total CA + CA-SP1 is presented in the graphs. The gel image shown here is representative of three independent experiments. **b** TZM-bl cells were infected with p24 normalized HIV-1 subtype C viruses K3016 WT and its mutants, SP1:N9S, SP1:G10T and SP1:N9S/G10T produced from MI treated HEK-293T cells for 48 h to measure infectivity. Quantitative data for levels of infectivity relative to the DMSO control-treated sample is shown. Error bars indicate standard deviations from three independent experiments. The asterisks indicate significant differences (*p < 0.05, **p < 0.01, ***p < 0.001, ****p < 0.0001) and ns indicates not significant

**Fig. 9:**
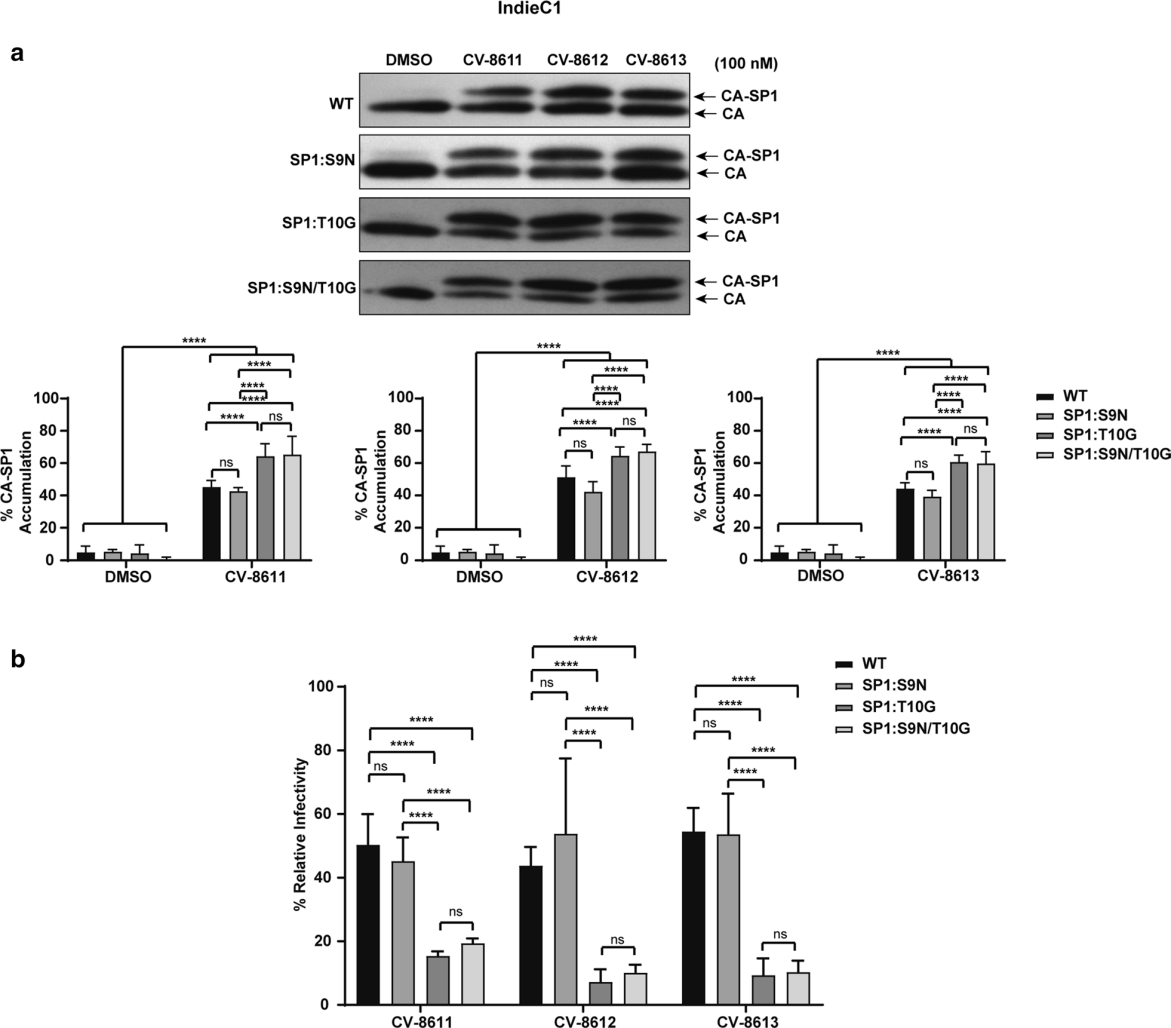
Mutation in HIV-1 subtype C IndieC1 Gag-SP1 residue 10 increases sensitivity towards BVM analogs. **a** HEK-293T cells were transfected with the HIV-1 subtype C clones, IndieC1 WT and its mutants SP1:S9N, SP1:T10G and SP1:S9N/T10G. Cells were treated with 100 nM of BVM analogs CV-8611, CV-8612, CV-8613 or with DMSO only. The virion-associated CA and CA-SP1 were detected by immunoblotting. Quantification of % CA-SP1 relative to total CA + CA-SP1 is presented in the graphs. The gel image shown here is representative of three independent experiments. **b** TZM-bl cells were infected with p24 normalized HIV-1 subtype C viruses IndieC1 WT and its mutants SP1:S9N, SP1:T10G and SP1:S9N/T10G produced from MI treated HEK-293T cells for 48 h to measure infectivity. Quantitative data for levels of infectivity relative to the DMSO control-treated sample is shown. Error bars indicate standard deviations from three independent experiments. The asterisks indicate significant differences (*p < 0.05, **p < 0.01, ***p < 0.001, ****p < 0.0001) and ns indicates not significant

**Fig. 10:**
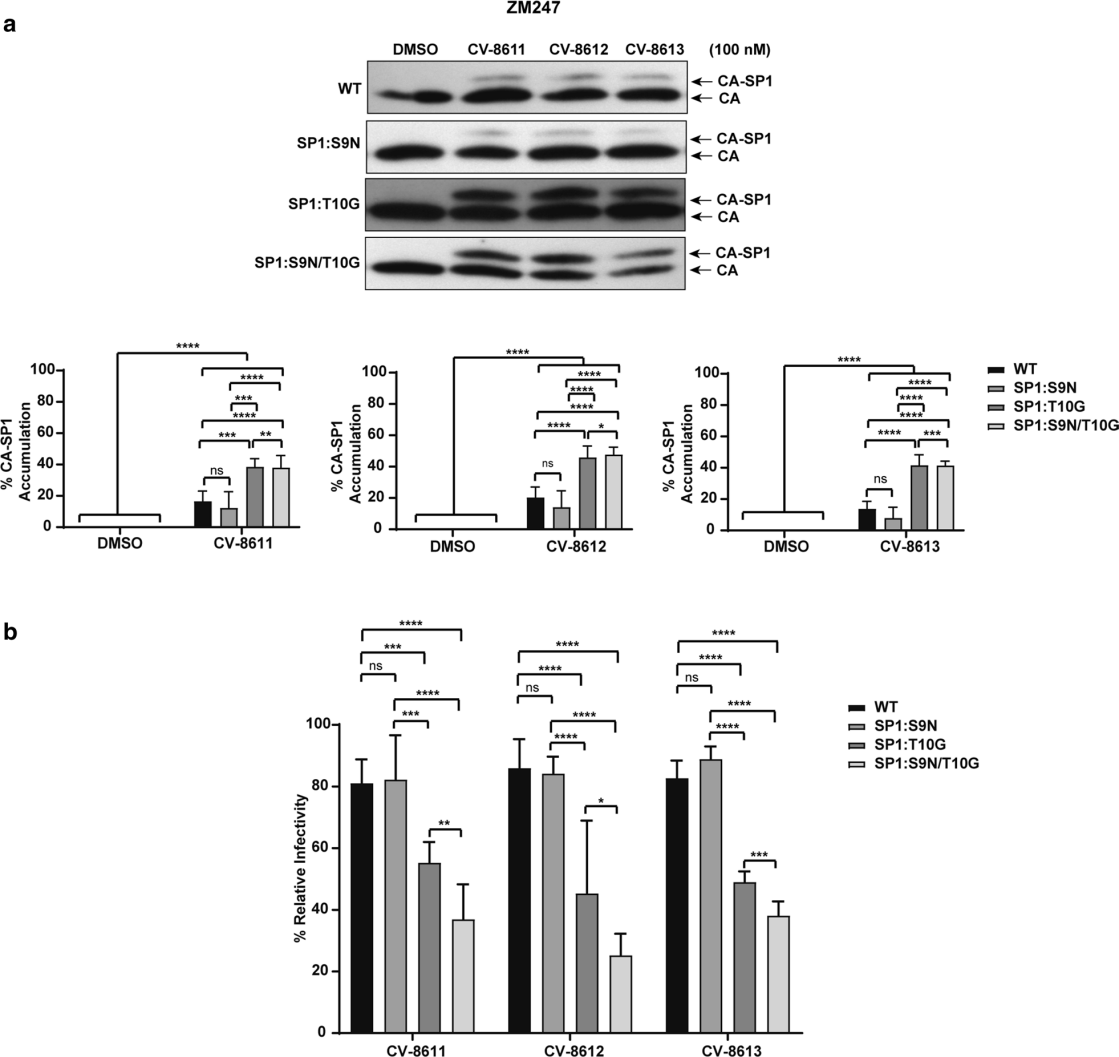
Mutation in HIV-1 subtype C ZM247 Gag-SP1 residue 10 increases sensitivity towards BVM analogs. **a** HEK-293 T cells were transfected with the HIV-1 subtype C clones, ZM247 WT and its mutants SP1:S9N, SP1:T10G and SP1:S9N/T10G. Cells were treated with 100 nM of BVM analogs CV-8611, CV-8612, CV-8613 or with DMSO only. The virion-associated CA and CA-SP1 were detected by immunoblotting. Quantification of % CA-SP1 relative to total CA + CA-SP1 is presented in the graphs. The gel image shown here is representative of three independent experiments. **b** TZM-bl cells were infected with p24 normalized HIV-1 subtype C viruses ZM247 WT and its mutants SP1:S9N, SP1:T10G and SP1:S9N/T10G produced from MI treated HEK-293T cells for 48 h to measure infectivity. Quantitative data for levels of infectivity relative to the DMSO control-treated sample is shown. Error bars indicate standard deviations from three independent experiments. The asterisks indicate significant differences (*p < 0.05, **p < 0.01, ***p < 0.001, ****p < 0.0001) and ns indicates not significant

The efficacy of the BVM analog CV-8611 on the WT (K3016, IndieC1 and ZM247) and the mutant viruses (SP1:9, SP1:10 and SP1: 9/10) was analyzed by performing the CA-SP1 accumulation (Figs. 8, 9, 10a) and single cycle infectivity assay (Figs. 8, 9, 10b). Swapping glycine with a threonine at the residue 10 in K3016 SP1: G10T reduced the accumulation of the CA-SP1 intermediate by 2.5 fold in the presence of the BVM analog CV-8611 whereas replacing asparagine with a serine at SP1 residue 9 in K3016 SP1: N9S had negligible effect as compared to the WT. The double mutant K3016 SP1: N9S/G10T displayed similar sensitivity as the single mutant SP1:G10T (Fig. 8a). Similar results were observed in presence of BVM analogs CV-8612 and CV-8613 (Fig. 8a). These results suggested that the glycine residue at SP1 position 10 may play a major role in determining the sensitivity of the K3016 virus to the BVM analogs. Similar assays were performed using IndieC1 and ZM247 WT viruses and their mutants (SP1:S9N, SP1:T10G and SP1:S9N/T10G). Replacing threonine with a glycine (SP1: T10G) in both IndieC1 and ZM247 markedly increased the CA-SP1 accumulation by nearly 2-fold in the presence of the BVM analog CV-8611 whereas mutating the residue 9 (SP1: S9N) had no effect as compared to the WT virus (Figs. 9a, 10a). The double mutant SP1: S9N/T10G also displayed a similar increase in the CA-SP1 accumulation as the single mutant SP1:T10G in the presence of CV-8611 (Figs. 9a, 10a). Similar results were observed in the presence of the other two BVM analogs (CV-8612 and CV-8613) (Figs. 9a, 10a).

The infectivity of the WT and the mutant viruses produced in the absence or the presence of the BVM analogs CV-8611, CV-8612 and CV-8613, was assayed as explained earlier in the Methods. The protocol of the infectivity assay and method of calculation of relative infectivity has been explained in a schematic diagram provided as Additional file 1: Fig. S1. Briefly, WT viruses (K3016, IndieC1 and ZM247) and their mutants SP1:9, SP1:10 and SP1:9/10 viruses were produced in presence and absence of 100 nM BVM analogs (CV-8611, CV-8612 and CV-8613) in HEK-293T cells. The viruses were quantified and normalized for p24. 5 ng of HIV-1 p24 equivalent virus was used to infect TZM-bl cells. 48 h post-infection, TZM-bl cells were lysed, luminescence was measured and relative infectivity was calculated as depicted in the Additional file 1: Fig. S1. The infectivity for each HIV-1 virus produced in the absence of BVM analogs (DMSO only control) was considered as 100% and relative infectivity of the same virus produced in presence of BVM analogs (100 nM) was calculated. We observed a 3.5-fold increase in the infectivity of the single mutant K3016 SP1: G10T and the double mutant K3016 SP1: N9S/G10T as compared to the WT virus produced in presence of CV-8611 whereas the relative infectivity of the single mutant K3016 SP1: N9S increased by 1.5 fold only in the presence of the analog (Fig. 8b) which was consistent with the CA-SP1 accumulation assay (Fig. 8a).

The relative infectivity of the mutant IndieC1 carrying SP1: T10G and SP1: S9N/T10G mutation was drastically decreased by fivefold in comparison to the WT virus in the presence of the analog CV-8611 (Fig. 9b). In contrast, the SP1:S9N mutant showed no significant change in relative infectivity in the presence of CV-8611 (Fig. 9b). Similarly, the ZM247 SP1: T10G mutant also displayed a greater decrease in the infectivity (55%) than the SP1: S9N mutant as compared to the WT in the presence of the CV-8611. The relative infectivity of the double mutant ZM247 SP1: S9N/T10G further decreased in the presence of the BVM analog CV-8611 (Fig. 10b). Similar results were observed in the presence of the other two BVM analogs (CV-8612 and CV-8613) (Figs. 8b, 9b, 10b). These results suggested that replacing the threonine residue at SP1 position 10 in the IndieC1 and ZM247 viruses with a glycine imparted increased sensitivity of these viruses to the BVM analogs, while swapping the glycine with a threonine at the same position in the K3016 virus decreased its sensitivity to the BVM analogs. The important role played by the glycine at SP1 position 10 was further confirmed by measuring the antiviral activity of the WT and the mutant viruses as described in the Methods [38]. The IC_50_ values for K3016 SP1:G10T mutant increased drastically to > 500 nM from 2.55 nM (WT) indicating that the point mutation at SP1 residue 10 had decreased the sensitivity of the virus to the BVM analogs (Table 2). In contrast, replacing the threonine with a glycine at the same position in the IndieC1 and ZM247 viruses improved the IC_50_ values significantly confirming that the point mutation increased the sensitivity of the viruses to the BVM analogs (Table 2, compare WT and SP1:T10G mutants). Taken together, our results strongly suggested that polymorphism at residue 10 in the HIV-1 subtype C Gag-SP1 region played a crucial role in regulating the sensitivity of the HIV-1 subtype C viruses against the BVM analogs.

**Table 2:** Antiviral activities of the BVM analog CV-8611 against the WT and mutant HIV-1 subtype C viruses

Compound	IC_50_ (nM)
K3016 WT	2.55375 ± 0.699
K3016 SP1:G10T	> 500 nM
IndieC1 WT	133.6145 ± 2.59
IndieC1 SP1:T10G	31.3055 ± 9.586
ZM247 WT	> 500 nM
ZM247 SP1:T10G	116.9423 ± 6.893

The IC_50_s represent the mean ± standard deviations from two independent experiments

## Discussion

Several studies have reported that the BVM analogs containing chemical modifications at the C-3, C-28 or C-30 positions were more potent than the parental compound BVM against multiple HIV-1 subtypes including HIV-1 subtype C [16, 17, 28, 29, 39]. The current study primarily focused on analyzing the effect of three novel C-28 alkyl amine derivatives of BVM (CV-8611, CV-8612, CV-8613) against both HIV-1 subtype B and C strains. All analogs reduced the CA-SP1 cleavage and the infectivity of the HIV-1 subtype B, NL4-3 and the subtype C K3016 virus (Figs. 1, 2).

Resistance selection experiments performed by culturing the K3016 virus in the presence of the BVM analogs led to the emergence of two mutations, SP1: A1V and CA: I120V. The SP1: A1V was the most prevalent mutation that conferred resistance against all the MIs tested [28, 30, 36, 38, 40]. According to the Los Alamos HIV sequence database, Alanine (SP1 residue 1) was highly conserved and found in 99.63% virus isolates with valine present in only 0.017% virus isolates. Whereas, isoleucine (CA residue 201) was highly conserved (present in 99.66% of viral sequences of all HIV-1 subtypes) [23]. This analysis suggested that these residues were highly conserved and mutations obtained were not present naturally but induced in the presence of the compounds [23, 41].

The crystal structure of HIV-1 subtype B CTD-SP1 Gag fragment is a goblet-shaped hexamer in which CTD forms the cup followed by a type II β-turn, and the stem region of a 6-helix bundle contains the CA-SP1 junction [13, 14, 42]. The CA-SP1 cleavage site is buried inside the six-helix bundle and unfolds for protease to gain access to this site. MI binding may stabilize the six-helix bundle, thereby limiting protease access and blocking CA-SP1 processing. However, the mutation at SP1-A1V may confer resistance by destabilizing Gag thus making the CA-SP1 cleavage site accessible to the viral protease. More accessibility to the viral protease may increase the rate of CA-SP1 cleavage which may eventually inhibit the action of MI [38, 43, 44] and result in conferring MI resistance. Neyret et al., reported that SP1:A1V mutation in HIV-1 subtype B NL4-3 virus suppressed the interaction of a BVM analog EP-39 with CA-SP1(A1V)-NC Gag peptide in comparison to the WT [28]. Hence, it is possible that the K3016 SP1: A1V mutation may confer resistance to the compound CV-8612 in a similar manner.

The second mutant K3016 CA:I201V was identified in the presence of BVM analog CV-8613 and conferred partial resistance and compound dependence on the HIV-1 subtype C virus consistent with our previous study using another MI, PF-46396 [37]. In contrast, the HIV-1 subtype B CA:I1201V mutant conferred resistance but not compound dependence against MIs: GSK3532795 and PF-46396 [36, 39]. This phenotype of the HIV-1 subtype C CA:I201V mutant reflected a unique characteristic of this virus [37]. It is interesting to note that mutation at this residue did not impair virus assembly, but reduced infectivity and replicative fitness which was markedly improved on addition of BVM analog CV-8613 (Fig. 5b, c). In the primary structure of Gag, CA 201 is located distal to the CA- SP1 junction. In the immature HIV-1 Gag lattice, the helix 9/10 loop containing CA 201 residue is proximal to the CA-SP1 junction helix and may be involved in the formation of the CA hexagonal lattice as well as in the interaction with MIs [13]. Hence, CA: I201V mutation may cause a conformational change in the immature Gag lattice which may lead to an increase in the infectivity and replicative fitness of the virus in the presence of BVM analog.

In comparison to HIV-1 K3016, studies against two other HIV-1 subtype C molecular clones, IndieC1 and ZM247, revealed a reduced sensitivity of these viruses towards the BVM analogs with ZM247 being the least sensitive. Sequence analysis of the three HIV-1 subtype C clones revealed a 94% similarity in the CA-CTD region. It has been well established that the CA-CTD and the SP1 regions of Gag are crucial for virus maturation [6, 22, 45]. An analysis of the sequences of the CA-CTD region from the three viral clones, revealed three residues (CA: 155, CA: 200, CA: 204) that were identical in IndieC1 and ZM247 but distinct in K3016. However, none of these CA-CTD polymorphs have been previously reported to be crucial for resistance against MIs. An alignment of the sequences in the SP1 region of the three HIV-1 subtype C viruses identified polymorphisms at residue 9 and 10 with an asparagine and a glycine present in K3016 and a serine and a threonine in IndieC1 and ZM247, respectively. Polymorphisms at the SP1 residue 9 have not been previously reported to be associated with resistance to MIs. In our current study, changes in SP1:9 did not significantly alter the MI phenotype of the mutant virus. Mutations in SP1:10 in K3016 had conferred partial resistance to the BVM derivative 7m [38]. In our current study, we observed a strong association between the SP1:10 polymorphism and sensitivity of the viruses to BVM analogs. Replacing the glycine at SP1 residue 10 had a drastic impact on the MI sensitivity of K3016 while substituting the asparagine at residue 9 showed no significant impact. Similarly, introduction of a glycine at SP1 position 10 in IndieC1 and ZM247 significantly increased their sensitivity to the BVM analogs, whereas swapping the serine with an asparagine at SP1 position 9 did not alter the drug resistance phenotype of the virus. However, it is important to note that among 1194 HIV-1 subtype C viruses in the curated Los Alamos database, 65% have asparagine and 20% have serine at SP1:9; 25.61% contain threonine at SP:10; whereas only 4.89% have glycine (Fig. 7b). Thus, the glycine residue that conferred resistance to the BVM analogs was the least prevalent among the HIV-1 subtype C viruses. Hence, there may be a greater propensity for the majority of HIV-1 subtype C viruses to exhibit a degree of resistance to the BVM analogs. Although the amino acid position 9 and 10 are highly polymorphic, spontaneous mutations have not been reported in these positions in the absence of drugs [41]. The role of polymorphism at SP1 residue 10 in regulating the activity of BVM analogs appeared to be subtype specific as mutating the corresponding residue in the HIV-1 subtype B clone, NL4-3 did not alter the sensitivity of the NL4-3 virus to the BVM analogs (data not shown).

Although several structural studies have resolved the CA-CTD-SP1 junction, none of them have included the SP1 residues beyond the 8^th^ amino acid [14, 42, 46, 47, 49]. This could be due to the highly dynamic nature of SP1 residues [46–49]. The structure and dynamics analysis of the CA_CTD_- SP1-NC region has revealed that the glycine at SP1 residue 10 may be present in the H6 junction helix [48] and may stabilize the immature Gag lattice through hydrophobic interactions with the MIs. Substitution of the glycine with the hydrophilic threonine may inhibit this interaction, allowing access of the CA-SP1 site to protease mediated cleavage.

Further structural studies including the entire SP1 region would help in elucidating the important role of SP1 residue 10 in regulating sensitivity to MIs.

Although, we can predict the probable mechanism of action of the MIs on the basis of available structure of HIV-1 subtype B CA-CTD-SP1 region [13, 14, 42, 46, 47, 49], structural studies with HIV-1 subtype C Gag would immensely help in elucidating the basis for the phenotypic differences between the two HIV-1 subtypes. In addition to cleavage site mutations, the distal effects caused by the non-cleavage site mutations are indispensable for the Gag function and replication. Conformational changes occurring in the Gag protein due to the non-cleavage sites mutations conferred resistance to protease inhibitors [50, 51]. Another study highlighted the importance of analysing protease inhibitor susceptibility in the context of full-length Gag [52]. Structural insights into the role of resistant mutations will aid in the design of novel Gag inhibitors. While these mutations may arise in different residues in the Gag, they may be close to each other in the overall structure of Gag. Hence screening the whole Gag structure for the probable allosteric drug targeting sites can be an approach to identify novel drugs. However, non-availability of structure of full-length Gag is a major limitation. The structural studies may help in designing a common multi-cleavage site inhibitor to target multiple sites at the same time [53, 54] or different drugs may be used in combination to target multiple sites [53]. Such a strategy would delay drug resistance. An *in-vitro* CA-SP1 cleavage assay can be utilized to screen the resistant mutants, which can be helpful in rational designing of novel drugs [43]. Furthermore, capsid protein can be targeted with highly potent and potentially long-acting capsid inhibitors along with MIs [55].

Hence, taken together our study has highlighted the importance of studying polymorphisms in the HIV-1 subtype C SP1 region in order to develop potent and broadly active Maturation inhibitors and further emphasized that structural studies of CA-SP1 region of HIV-1 subtype C Gag would immensely help in designing of novel inhibitors.

## Conclusions

HIV-1 subtype C contributes to nearly 50% of the total HIV infections world-wide. To the best of our knowledge, our study has highlighted for the first time, the crucial role of polymorphisms in the SP1 region of the HIV-1 subtype C Gag in regulating sensitivity towards the BVM analogs. We have identified that the glycine residue present at the HIV-1 Gag SP1 position 10 imparted a higher sensitivity to the HIV-1 subtype C viruses against the BVM analogs. These studies will aid in rational design of novel MIs with high efficacy, low toxicity and broad specificity which may prove efficacious in clinics.

## Methods

### Preparation of compounds

BVM analogs used in the study were synthesized by modifications at C-28 positions of Bevirimat (BVM), as described in Urano et al*.* [16]. These BVM analogs were synthesized by DFH Pharma. Compounds CV-8611, CV-8612, and CV-8613 used in the present study were dissolved in dimethyl sulfoxide (DMSO) and stored in the dark at − 80 °C.

### Cell culture, plasmids, and transfections

HEK-293T and HUT-R5 T-cells were kind gifts from Dr. Eric O. Freed, National Cancer Institute, NIH, USA. TZM-bl cells (catalog number: 8129) were obtained through the NIH AIDS Reagent Program, Division of AIDS, NIAID, NIH. HEK-293T and TZM-bl cells were cultured in complete Dulbecco’s modified Eagle’s medium (DMEM). HUT-R5 T-cells were maintained in Roswell Park Memorial Institute (RPMI)-1640 medium supplemented with 10% fetal bovine serum (FBS), 100 U/ml penicil lin, and 100 µg/ml streptomycin. HIV-1 subtype B clone NL4-3 (a kind gift from Dr Eric O. Freed, National Cancer Institute, NIH, USA; GenBank accession: AF324493.2), HIV-1 subtype C clones; K3016 (South African origin, a kind gift from Dr. Christina Ochsenbauer, University of Alabama, USA; GeneBank accession: KC156129), ZM247 (Zambian origin, a kind gift from Dr Eric O. Freed, National Cancer Institute, NIH, USA; GeneBank accession: FJ496200.1) and IndieC1 (Indian origin, a kind gift from Dr. Uday Ranga, JNCASR, India; GeneBank accession: AB023804) were used in this study. Plasmid DNAs were purified using Thermo Fisher Scientific Plasmid Maxiprep kit as per manufacturer’s instructions. HEK-293 T cells were transfected with HIV-1 DNAs (HIV-1 subtype B clone NL4-3, HIV-1 subtype C clone K3016 WT and its mutants SP1: A1V, SP1:I201V, SP1:N9S, SP1:G10T, SP1:N9S/G10T, HIV-1 subtype C clone IndieC1 WT and its mutants SP1:S9N, SP1:T10G, SP1:S9N/T10G, HIV-1 subtype C clone ZM247 WT and its mutants SP1:S9N, ZM247 SP1:T10G, ZM247 SP1:S9N/T10G) (3 µg) using Lipofectamine 2000 (Invitrogen catalog number: 11668-019) following manufacturer’s recommendations, whereas HUT-R5 T-cells were transfected with HIV-1 DNAs using DEAE-dextran as described previously [56].

### CA-SP1 accumulation assays

CA-SP1 accumulation assays were performed as described previously [17]. Briefly, HEK-293T cells were transfected with HIV-1 DNAs (HIV-1 subtype B clone NL4-3 WT, HIV-1 subtype C clone K3016 WT and its mutants SP1: A1V CA:I201V, SP1:N9S, SP1:G10T and SP1:N9S/G10T, HIV-1 subtype C clone IndieC1 WT and its mutants SP1:S9N, SP1:T10G, and SP1:S9N/T10G, HIV-1 subtype C clone ZM247 WT and its mutants SP1:S9N, SP1:T10G and SP1:S9N/T10G), as mentioned above. 24 h post-transfection, the culture medium was replaced with fresh DMEM and incubated for an additional 2 h. Maturation inhibitors (MIs) were maintained in the culture throughout transfection. The culture supernatant was centrifuged at 845 × *g* for 3 min to remove cellular debris and then filtered using a 0.45 μm pore size filter and stored at – 80 °C as virus stocks. For the CA-SP1 accumulation assay, the 2 h viral supernatant was pelleted by ultracentrifugation at 210,100 × *g* for 1 h at 4 °C using SW41Ti rotor (Beckman Coulter, USA). The virus pellet was resuspended in 1 × radioimmunoprecipitation assay (RIPA) buffer (50 mM Tris–HCl pH 8.0, 150 mM sodium chloride, 1.0% NP-40, 0.5% sodium deoxycholate, 0.1% SDS) containing 1 × protease inhibitor cocktail (Roche, Germany catalog number 11697498001). The viral lysates were subjected to SDS–polyacrylamide gel electrophoresis (15% gel); proteins were transferred to polyvinylidene difluoride (PVDF) membrane and incubated with HIV-IgG obtained from the NIH/AIDS Reagent Program (catalog no. 3957) followed by incubation with HRP-conjugated anti-human secondary antibodies (GE Healthcare, catalog number: NA933). The proteins were visualized by enhanced chemiluminescence (Pierce, USA), and the bands were quantified using ImageJ software (http://imagej.nih.gov/ij/).

### Virus replication assays

Virus replication assays were performed as described previously [37]. Briefly, 5 × 10^6^ HUT-R5 T-cells were transfected with 5 µg HIV-1 DNAs and maintained in the absence or presence of MIs. Cells were split in the ratio 2:1 on every third day. Virus replication kinetics was monitored by quantifying p24 antigen using the HIV-1 p24 Antigen Capture kit (ABL, USA catalog number 5447) by following manufacturer’s recommendations. To identify mutations that conferred resistance to the compounds, the cell pellets were collected on the days of peak HIV-1 p24 concentrations, and genomic DNAs were extracted using a Blood DNA extraction kit (Qiagen, catalog number: 51104). The entire Gag-coding region of the provirus was PCR amplified and sequenced.

### Site-directed mutagenesis

Mutations were done on the K3016 WT, IndieC1 WT and ZM247 WT plasmid DNA backbone with synthetic complementary oligonucleotides (IDT, Belgium) using the QuickChange II XL site-directed mutagenesis kit (Agilent Technologies, catalog no: 200521) as per manufacturer’s instructions. Synthetic complementary oligonucleotides used were: K3016 SP1: A1V; 5′-GGCAAGGGTGTTGGTTGAGGCAATGAGCC-3′, K3016 CA: I201V; 5′-GCGAACCCAGATTGTAAGATCGTTTTAAGAGGATTAGGACC-3′, K3016 SP1:N9S 5′-GCTGAGGCAATGAGCCAAGCAAACAGTGGAAACATAATG-3′,K3016 SP1: G10T 5′-CTCTGCATCATTATGTTTGTATTGTTTGCTTGGCTCATTGCCTC-3′, K3016 SP1: N9S/G10T 5′-GGCAATGAGCCAAGCAAACAGTACAAACATAATGATGCAGA-3′, IndieC1 SP1: S9N 5′-CAATGAGCCAAGCAAACAATACCATACTGATGCAGAG-3′, IndieC1 SP1: T10G 5′-GCTTCTCTGCATCAGTATGCCACTGTTTGCTTGGCTCATT-3′, IndieC1 SP1: S9N/G10T 5′-TGAGGCAATGAGCCAAGCAAACAATGGCATACTGAT-3′, ZM247 SP1: S9N 5′-GCAATGAGCCAAGCAAACAACACAAACATAATGATGCAG-3′, ZM247 SP1: T10G 5′-TTTCTCTGCATCATTATGTTTCCGCTGTTTGCTTGGCTCATTGC-3′, ZM247 SP1: S9N/T10G 5′-GCTGAGGCAATGAGCCAAGCAAACAACGGAAACATAATG-3′. The change from the WT DNA sequence is underlined. Mutations were confirmed by DNA sequencing.

### Antiviral assays

HUT-R5 T-cells were infected with normalized HIV-1 subtype C K3016 virus (50 ng p24 equivalent) stocks at 37 °C for 2 h. Cells were then cultured in the presence of serial dilutions of each compound. After 8 days, the virus supernatants were collected, and the HIV-1 p24 concentration was quantified in the virus supernatants. The 50% inhibitory concentrations (IC_50_s) were calculated as the concentrations of MI that reduced HIV-1 p24 concentrations to 50% relative to DMSO-only controls.

IC_50_ analyses using single-cycle infectivity assays were also performed [38]. HEK-293T cells were transfected K3016 WT or its mutants-SP1:N9S, SP1:G10T, SP1:N9S/G10T, IndieC1 WT or its mutants- SP1:S9N, SP1:T10G, SP1:S9N/T10G, ZM247 WT or its mutants-SP1:S9N, SP1:T10G, SP1:S9N/T10G, and cultured in the presence of serial dilution of the BVM analog. Virus-containing supernatants were harvested, normalized for HIV-1 p24 concentration, and used to infect TZM-bl cells. Luciferase activity was measured at 2 days post infection and infectivity was calculated. The 50% inhibitory concentrations (IC_50_s) were calculated as the concentrations of MI that reduced infectivity to 50% relative to DMSO-only controls.

### Viral infectivity assay

Single-round infectivity assays were performed as previously described [57]. HEK-293T cells were transfected with HIV-1 DNAs (HIV-1 subtype B clone NL4-3, HIV-1 subtype C clone K3016 WT and its mutants SP1: A1V, SP1:I201V, SP1:N9S, SP1:G10T, SP1:N9S/G10T, HIV-1 subtype C clone IndieC1 WT, and its mutants SP1:S9N, SP1:T10G, SP1:S9N/T10G, HIV-1 subtype C clone ZM247 WT, and its mutants SP1:S9N, SP1:T10G, SP1:S9N/T10G as mentioned above. Either DMSO or maturation inhibitors (MIs) were maintained in the culture throughout transfection. 24 h post-transfection, culture supernatant was collected and centrifuged at 845 × *g* for 3 min to remove cellular debris and filtered using a 0.45 μm pore size filter disc to remove residual cellular contaminants and stored at – 80 °C as virus stocks. The virus stocks were normalized for p24 antigen using an HIV-1 p24 Antigen Capture kit (ABL, USA catalog number 5447). 5 ng HIV-1 p24 equivalent virus was used to infect TZM-bl cells (5 × 10^4^/well) in the presence of 20 µg DEAE-dextran per ml in 24 wells plate. The luciferase activity in the cell lysates was measured 48 h post-infection using the Steady-Glo luciferase assay kit (Promega catalog number: E2510) following the manufacturer’s recommendations. The schematic diagram for the detail methodology is given as Additional file 1: Fig. S1.

### Statistics

For statistical analysis, one-way ANOVA was done by implying Tukey’s test. *p*-values below 0.05, 0.01,0.001 and 0.0001 were denoted as *, **, *** and ****, respectively. Non-significant results were denoted as ns.

## Supplementary Information

**Additional file 1: Figure S1. **Schematic diagram representing the methodology of infectivity assay and calculation of relative infectivity. A) HEK-293 T cells were seeded in a 6-wells plate, grown to 60% confluency and transfected with the HIV-1 subtype C clones K3016 WT and its mutants, SP1: N9S, SP1: G10T and SP1: N9S/G10T using Lipofectamine 2000. Cells were treated with 100 nM of BVM analog CV-8611or with DMSO only. 24 h post-transfection, the virus was collected, centrifuged to clarify cell debris, filtered through 0.45 μm syringe filter. The HIV-1 p24 antigen was quantified by ELISA. B) 5X10^4^ TZM-bl cells/well were seeded in 24-wells plate. After 24 h, 5 ng HIV-1 p24 equivalent virus was used to infect TZM-bl cells. C) 48 h post-infection, cells were lysed using Glo Lysis buffer, Steady Glo substrate was added and luminescence was measured. For each well, two readings were measured. Background luminescence unit from mock infected cells was deducted from all the values. Average luminescence unit was calculated from two readings taken from a single sample. Infectivity of the HIV-1 virus produced in DMSO only control was taken as 100% infectivity and relative infectivity of the virus produced in presence of BVM analog CV-8611 was calculated. The same procedure was repeated for HIV-1 subtype C Indie C1 and ZM247 using all three BVM analogs.

## Data Availability

The data used in this study are available from the corresponding author based on reasonable request.

## Abbreviations

A: Alanine
AIDS: Acquired immunodeficiency syndrome
ANOVA: Analysis of variance
ART: Antiretroviral therapy
BVM: Bevirimat
CA: Capsid
CTD: Carboxy terminal domain
DEAE: Diethylethanolamine
DMSO: Dimethyl sulfoxide
FDA: Food and Drug Administration
G: Glycine
HIV: Human Immunodeficiency virus
HRP: Horseradish peroxidase
I: Isoleucine
IC_50_: 50% Inhibitory concentration
IgG: Immunoglobulin G
MA: Matrix
MI: Maturation inhibitor
N: Asparagine
NC: Nucleocapsid
nM: Nanomolar
NNRTI: Non-nucleoside reverse transcriptase inhibitor
NRTI/NtRTI: Nucleoside/nucleotide reverse transcriptase inhibitors
PR: Protease
PVDF: Polyvinylidene difluoride
QVT: Glutamine valine threonine
S: Serine
SDS-PAGE: Sodium dodecyl sulphate–polyacrylamide gel electrophoresis
SP1: Spacer peptide 1
T: Threonine
V: Valine
WT: Wild type
