# Supplementary figures and images for: A single G10T polymorphism in HIV-1 subtype C Gag-SP1 regulates sensitivity to maturation inhibitors
Source: Retrovirology. 2021 Apr 9;18:9. doi: 10.1186/s12977-021-00553-5 (PMC8033686; doi:10.1186/s12977-021-00553-5)

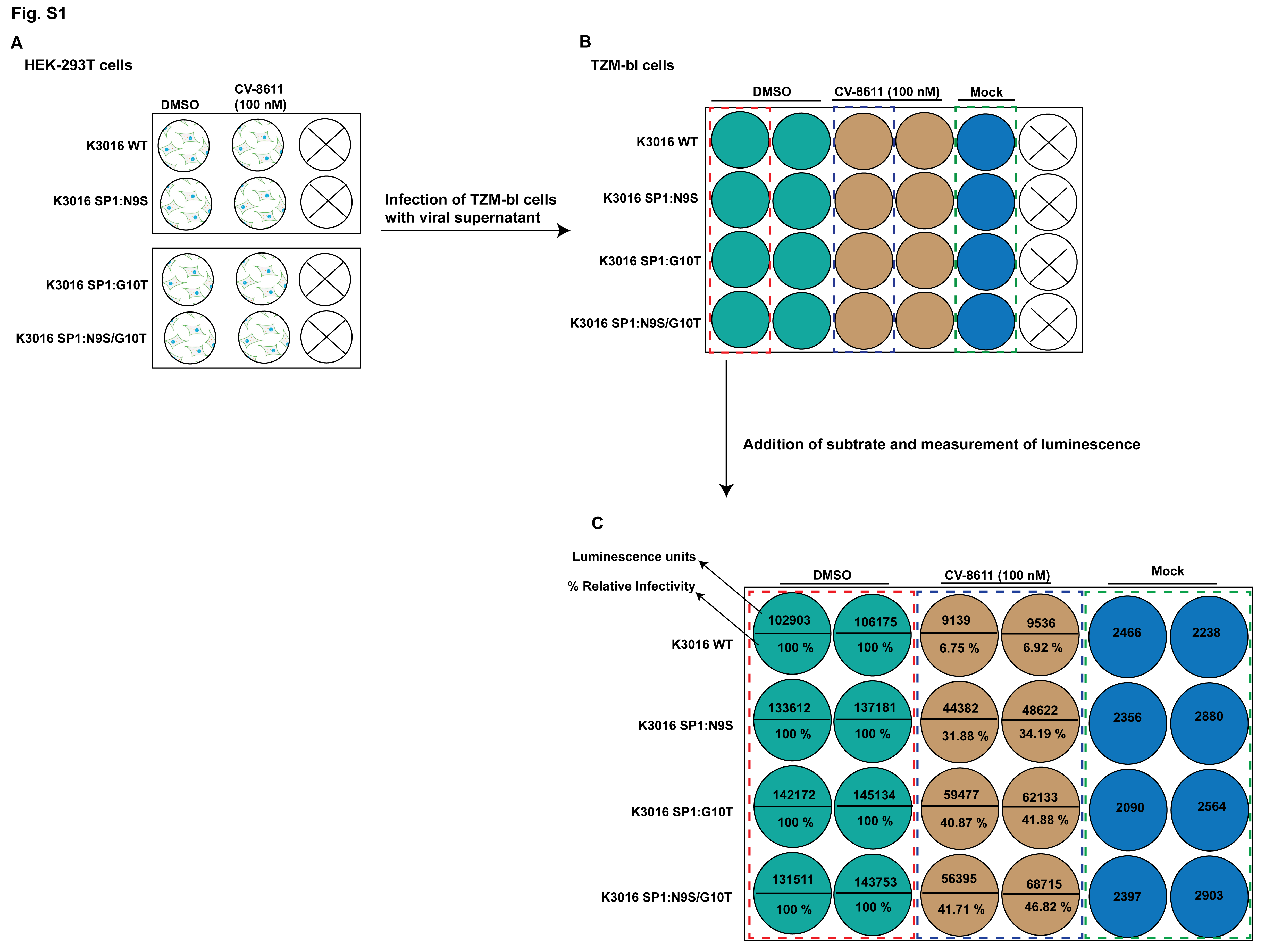

Supplement: Supplementary file 1 — Additional file 1: Figure S1. Schematic diagram representing the methodology of infectivity assay and calculation of relative infectivity. A) HEK-293 T cells were seeded in a 6-wells plate, grown to 60% confluency and transfected with the HIV-1 subtype C clones K3016 WT and its mutants, SP1: N9S, SP1: G10T and SP1: N9S/G10T using Lipofectamine 2000. Cells were treated with 100 nM of BVM analog CV-8611or with DMSO only. 24 h post-transfection, the virus was collected, centrifuged to clarify cell debris, filtered through 0.45 μm syringe filter. The HIV-1 p24 antigen was quantified by ELISA. B) 5X104 TZM-bl cells/well were seeded in 24-wells plate. After 24 h, 5 ng HIV-1 p24 equivalent virus was used to infect TZM-bl cells. C) 48 h post-infection, cells were lysed using Glo Lysis buffer, Steady Glo substrate was added and luminescence was measured. For each well, two readings were measured. Background luminescence unit from mock infected cells was deducted from all the values. Average luminescence unit was calculated from two readings taken from a single sample. Infectivity of the HIV-1 virus produced in DMSO only control was taken as 100% infectivity and relative infectivity of the virus produced in presence of BVM analog CV-8611 was calculated. The same procedure was repeated for HIV-1 subtype C Indie C1 and ZM247 using all three BVM analogs. [file 12977_2021_553_MOESM1_ESM.tif]
